# A Novel Multivariate Index for Pancreatic Cancer Detection Based On the Plasma Free Amino Acid Profile

**DOI:** 10.1371/journal.pone.0132223

**Published:** 2015-07-02

**Authors:** Nobuyasu Fukutake, Makoto Ueno, Nobuyoshi Hiraoka, Kazuaki Shimada, Koichi Shiraishi, Nobuhiro Saruki, Toshifumi Ito, Minoru Yamakado, Nobukazu Ono, Akira Imaizumi, Shinya Kikuchi, Hiroshi Yamamoto, Kazuhiro Katayama

**Affiliations:** 1 Department of Hepatobiliary and Pancreatic Oncology, Osaka Medical Center of Cancer and Cardiovascular Diseases, Osaka, Japan; 2 Division of Hepatobiliary and Pancreatic Medical Oncology, Kanagawa Cancer Center, Kanagawa, Japan; 3 Division of Pathology and Clinical Laboratories, National Cancer Center Hospital, Tokyo, Japan; 4 Hepatobiliary and Pancreatic Surgery Division, National Cancer Center Hospital, Tokyo, Japan; 5 Division of Gastroenterology, Department of Internal Medicine, Tokai University Oiso Hospital, Kanagawa, Japan; 6 Department of Anesthesia, Gunma Prefectural Cancer Center, Gunma, Japan; 7 Department of Gastroenterology and Hepatology, Japan Community Healthcare Organization (JCHO), Osaka Hospital, Osaka, Japan; 8 Center for Multiphasic Health Testing and Services, Mitsui Memorial Hospital, Tokyo, Japan; 9 Institute for Innovation, Ajinomoto Co., Inc., Kanagawa, Japan; Centro Nacional de Investigaciones Oncológicas (CNIO), SPAIN

## Abstract

**Background:**

The incidence of pancreatic cancer (PC) continues to increase in the world, while most patients are diagnosed with advanced stages and survive <12 months. This poor prognosis is attributable to difficulty of early detection. Here we developed and evaluated a multivariate index composed of plasma free amino acids (PFAAs) for early detection of PC.

**Methods:**

We conducted a cross-sectional study in multi-institutions in Japan. Fasting plasma samples from PC patients (n = 360), chronic pancreatitis (CP) patients (n = 28), and healthy control (HC) subjects (n = 8372) without apparent cancers who were undergoing comprehensive medical examinations were collected. Concentrations of 19 PFAAs were measured by liquid chromatography–mass spectrometry. We generated an index consisting of the following six PFAAs: serine, asparagine, isoleucine, alanine, histidine, and tryptophan as variables for discrimination in a training set (120 PC and matching 600 HC) and evaluation in a validation set (240 PC, 28 CP, and 7772 HC).

**Results:**

Several amino acid concentrations in plasma were significantly altered in PC. Plasma tryptophan and histidine concentrations in PC were particularly low, while serine was particularly higher than that of HC. The area under curve (AUC) based on receiver operating characteristic (ROC) curve analysis of the resulting index to discriminate PC from HC were 0.89 [95% confidence interval (CI), 0.86–0.93] in the training set. In the validation set, AUCs based on ROC curve analysis of the PFAA index were 0.86 (95% CI, 0.84–0.89) for all PC patients versus HC subjects, 0.81 (95% CI, 0.75–0.86) for PC patients from stage IIA to IIB versus HC subjects, and 0.87 (95% CI, 0.80–0.93) for all PC patients versus CP patients.

**Conclusions:**

These findings suggest that the PFAA profile of PC was significantly different from that of HC. The PFAA index is a promising biomarker for screening and diagnosis of PC.

## Introduction

Pancreatic cancer (PC) is currently the eighth leading cause of cancer-related mortality, with an estimated 266,000 deaths worldwide in 2008 [1], and remains one of the most challenging malignancies to treat. The only potentially curative therapy is surgical resection; however, approximately 70% of cases initially present with advanced disease (stage III–IV), which cannot be cured by surgery. Advanced PC has a very poor prognosis, which is attributable to absence of early symptoms and useful screening methods, with a median survival period of 7.7 months for stage III and 2.5 months for stage IV disease[2]. Moreover, the 5-year survival rates are reportedly only 21.3% for local stage, 8.9% for regional stage, and 1.8% for distant stage [3].

Several tumor-associated antigens have been evaluated as potential prognostic factors for PC, including carcinoembryonic antigen (CEA) and carbohydrate antigen (CA) 19–9. CA19-9 is the most clinically useful diagnostic marker, with sensitivity of 79%–81% and specificity of 82%–90% in symptomatic patients, but its low positive predictive value makes it a poor marker for screening [4]. In addition, enhanced computed tomography (CT) and endoscopic ultrasound (EUS) are useful for the diagnosis of PC; however, these modalities are costly and potentially hazardous. Therefore, it is necessary to establish more effective screening methods for PC, particularly in the early stages of the disease. Amino acids are either ingested or endogenously synthesized and play essential physiological roles both as basic metabolites and metabolic regulators. Plasma free amino acids (PFAAs) present favorable targets of biomarkers because PFAA profiles are known to be influenced by metabolic variations in specific organ systems induced by specific diseases [5][6][7][8] [9]. Previous comprehensive metabolomic studies have often focused on changes in PFAA profiles [10][11][12][13]. Measurement of PFAA concentrations as possible marker of disease is also a more advantageous strategy for accurate and high-throughput analysis using mass spectroscopy than comprehensive metabolomics. Changes in PFAAs profiles are characteristic of several cancers; thus, the development of a multivariate index composed of these PFAAs could be used to better discriminate individual cancer types from healthy controls [14][15]. In the present study, we investigated the patterns of PFAA profiles, and then developed and validated a multivariate index for detection of PC.

## Materials and Methods

### Ethics statement

This study was conducted in accordance with the Declaration of Helsinki and the protocol was approved by the ethics committees of the Osaka Medical Center of Cancer and Cardiovascular Diseases, Kanagawa Cancer Center, the National Cancer Center Hospital, Tokai University Hospital, Gunma Prefectural Cancer Center, JCHO Osaka Hospital, Mitsui Memorial Hospital, Kameda Medical Center, and the Kanagawa Health Service Association. All subjects gave written informed consent before participation in this study. All clinical information was anonymized before data analysis.

### Subjects

PC patients (n = 360) included in this study were recruited from the Osaka Medical Center of Cancer and Cardiovascular Diseases, the Kanagawa Cancer Center, the National Cancer Center Hospital, Tokai University Hospital, and the Gunma Prefectural Cancer Center between 2007 and 2014. Patients with chronic pancreatitis (CP; n = 28) were recruited from JCHO Osaka Hospital between 2013 and 2014, while healthy control (HC) subjects (n = 8372) who underwent comprehensive health examination were recruited from Kanagawa Health Service Association, Kameda Medical Center (Makuhari Clinic), and Mitsui Memorial Hospital between 2008 and 2010. Over 95% of consecutive PC and CP cases and HCs agreed to provide consent during the study period. PC patients with the following characteristics were excluded: (1) simultaneously diagnosed with cancer in another organ, (2) hepatitis C, and (3) under treatment with anti-cancer agents. CP patients with the following characteristics were excluded: (1) diagnosed with cancer and (2) hepatitis C. The inclusion criteria of HC subjects were as follows: (1) no history of any cancer, and (2) no history of hepatitis C. PC stage was determined according to the Sixth Edition of the International Union Against Cancer (UICC) Tumor–Node–Metastasis (TNM) Classification of Malignant Tumors [16]

### Dataset preparation

Among the 360 PC patients, 120 PC patients obtained early in blood collection order were used as a training dataset. To prepare the HC subjects in the training data set, 600 of 8372 HC subjects were selected using propensity score matching based on gender and age distribution. The remaining 240 PC and all 28 CP patients and 7772 HC subjects were used as a validation dataset.

### Body mass index (BMI)

The height and weight of all subjects were measured. BMI was calculated as weight in kilograms divided by height in square meters. BMI values were categorized as follows: underweight (<18.50 kg/m^2^), normal weight (18.50–24.99 kg/m^2^), overweight (25.00–29.99 kg/m2), and obese (>30.00 kg/m^2^).

### PFAA analysis

After overnight fasting, blood samples (5 mL) were collected from antecubital veins into tubes containing ethylenediaminetetraacetic acid disodium salt as an anticoagulant and were immediately (<1 min) placed in ice water or an ice-cold cooling container (Forte Grow Medical Co., Ltd., Tochigi, Japan). Plasma was separated from the whole blood samples by centrifugation at 3,000 rpm and 4°C for 15 min and stored at −80°C until analysis. After thawing, the plasma samples were deproteinized using acetonitrile at a final concentration of 80% before measurement of amino acid concentrations by high-performance liquid chromatography (HPLC)–electrospray ionization (ESI)–mass spectrometry (MS) by precolumn derivatization. These analytical methods have been described elsewhere [17] [18][19]. Concentrations of the following 19 amino acids were measured and analyzed: alanine (Ala), arginine (Arg), asparagine (Asn), citrulline (Cit), glutamine (Gln), glycine (Gly), histidine (His), isoleucine (Ile), leucine (Leu), lysine (Lys), methionine (Met), ornithine (Orn), phenylalanine (Phe), proline (Pro), serine (Ser), threonine (Thr), tryptophan (Trp), tyrosine (Tyr), and valine (Val).

### Statistical analysis

#### Mean and standard deviation (SD)

The mean amino acid concentrations ± SDs were calculated to determine summarized PFAA profiles for both patients and controls.

#### Mann–Whitney *U*-test

The Mann–Whitney *U*-test was used to assess significant differences of PFAA concentrations between patients and controls.

#### Receiver–operator characteristic (ROC) analysis

ROC analysis was performed to determine the capabilities of uni- and multivariate analyses to discriminate between patients and controls. The patient labels were fixed as positive class labels. Therefore, an area under the ROC curve (AUC of ROC) value of <0.5 indicated that the amino acid level was lower in patients than controls, whereas an AUC of ROC value of >0.5 indicated that it was higher. The 95% confidence intervals (95% CI) of the AUC of ROC for the discrimination of patients based on amino acid concentrations and ratios was also estimated using the methods described by Hanley and McNeil [20].

#### Logistic regression analysis

Multivariate logistic regression analysis was performed to estimate the model discriminating PC patient from control subjects.

#### Model selection of PFAA index

The PFAA index was defined as a multivariate model using PFAA concentrations as variables. Logistic regression analysis with variable selection was performed to distinguish PC patients from the HC subjects. The maximum number of explanatory variables was restricted to less than seven to avoid potential multicollinearity. For model selection, the AUC of ROC was obtained after leave one out cross validation (LOOCV). In brief, one matched set composed of one PC patient and corresponding control subjects was omitted from the training data set, and the logistic regression model was calculated using the remaining samples to estimate coefficients for each amino acid. The function values for the left-out matched set were calculated based on this model. This process was repeated until every sample in the study data set had been left out once.

### Software

All statistical and multivariate analyses were performed using MATLAB (MathWorks, Natick, MA, USA) and Prism (GraphPad Software, Inc., San Diego, CA, USA) statistical software.

## Results

### Characteristics of patients and control subjects

**Fig 1:**
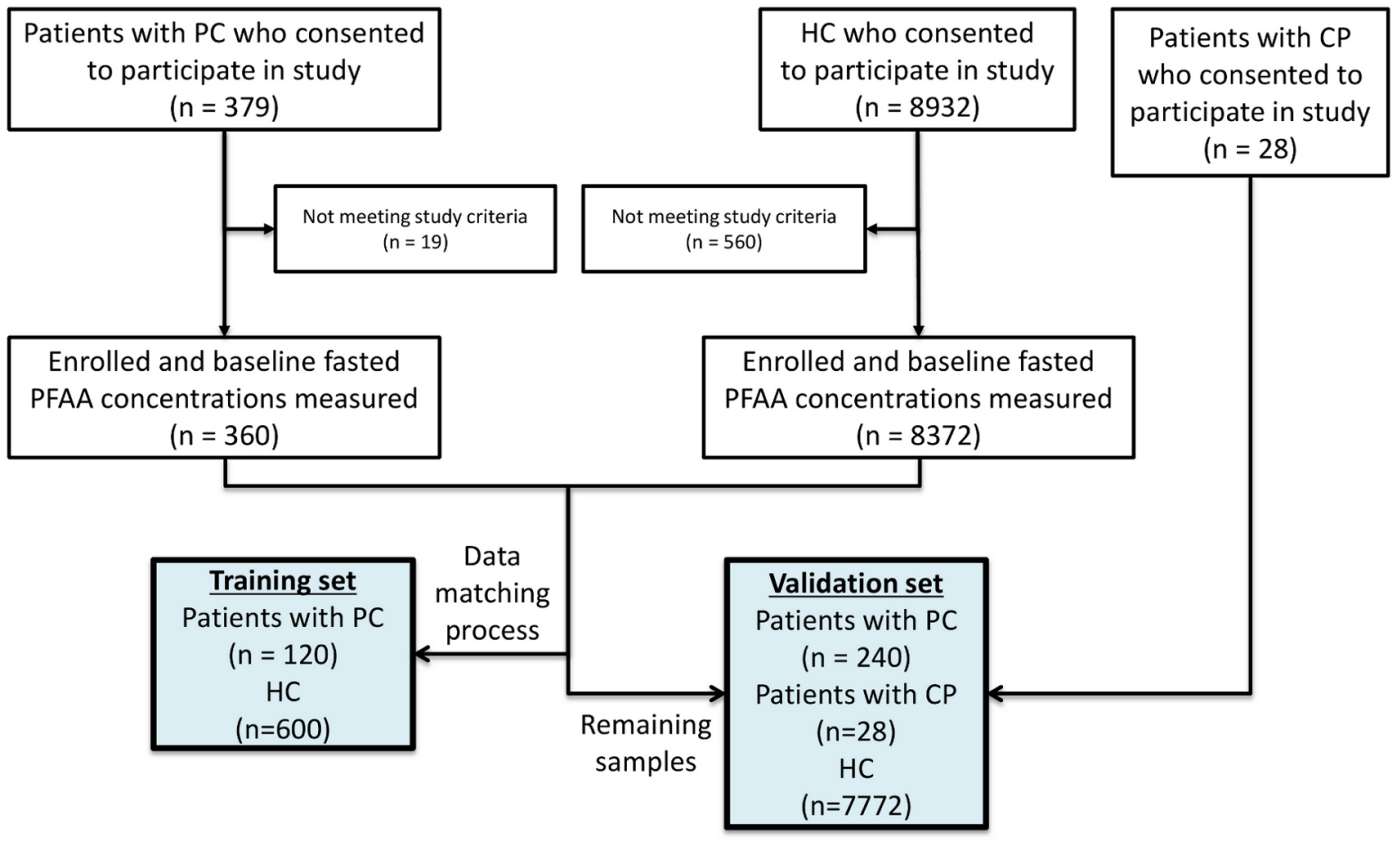
Summary of study design and inclusion and exclusion criteria.

Fig 1 shows an overview of this study. Fasting plasma samples were collected from the subjects (PC patients, n = 360; CP patients, n = 28; HC subjects, n = 8372). Table 1 summarizes the characteristics of PC and HC subjects included in this study. PC patients with stage 0–IIB disease, as a resectable stage subgroup, accounted for 35.8% of the training set and 35.0% of the validation set.

**Table 1:** Characteristics of PC patients and healthy controls.

		Training set			Validation set				
		PC (N = 120)	HC (N = 600)	p-value†	PC (N = 240)	HC (N = 7772)	p-value†	CP (N = 28)	p-value‡
**Gender**				0.15			0.79		0.55
Male	N (%)	65 (54.2)	367 (61.2)		151 (62.9)	4955 (63.8)		16 (57.1)	
Female	N (%)	55 (45.8)	233 (38.8)		89 (37.1)	2817 (36.2)		12 (42.9)	
**Age, Years**	Mean ± SD	66.2 ± 9.7*	64.3 ± 8.7		66.7 ± 9.1***	52.2 ± 10.0		61.5±13.7***	
	(range)	(30–87)	(29–89)		(41–85)	(20–88)		(31–81)	
**BMI, kg/m^2^**	Mean ± SD	21.0 ± 3.5***	22.8 ± 3.0	<0.001	21.4 ± 3.0***	22.7 ± 3.2	<0.001	21.3±2.6*	0.94
Underweight (<18.5)	%	22.5	5.8		16.3	8.1		14.3	
Normal range (18.5–24.9)	%	66.7	73.5		72.9	70.3		67.9	
Overweight (25.0–29.9)	%	9.2	18.7		10	19.3		10.7	
Obese (≥30.0)	%	1.7	2		0.8	2.3		0	
Missing	%	0	0		0	0		7.1	
**Smoking history**				0.54			0.04		0.02
Never	%	49.2	54.2		42.5	51.4		57.1	
Past	%	32.5	27.3		33.8	27.8		10.7	
Current	%	15	16.2		21.3	19.7		28.6	
Missing	%	3.3	2.3		2.5	1.1		3.6	
**Clinical Stages** ^a^	0	2	-		0	-		-	
	IA	3	-		0	-		-	
	IB	1	-		0	-		-	
	IIA	22	-		59	-		-	
	IIB	15	-		25	-		-	
	III	25	-		44	-		-	
	IV	52	-		104	-		-	
	Unknown	-	-		8	-		-	

Mann–Whitney U-test (versus HC),

*p < 0.05;

***p < 0.001

^**a**^: Cancer stages were determined according to the Union Internationalis Contra Cancrum (UICC) TNM Classification of Malignant Tumors, 6th Edition.

^†^ Chi-square tests to test the differences between PC and HC for categorical data of gender, BMI, and smoking history.

^‡^ Chi-square tests to test the differences between PC and CP for categorical data of gender, BMI, and smoking history.

### PFAA profiles of PC patients

**Fig 2:**
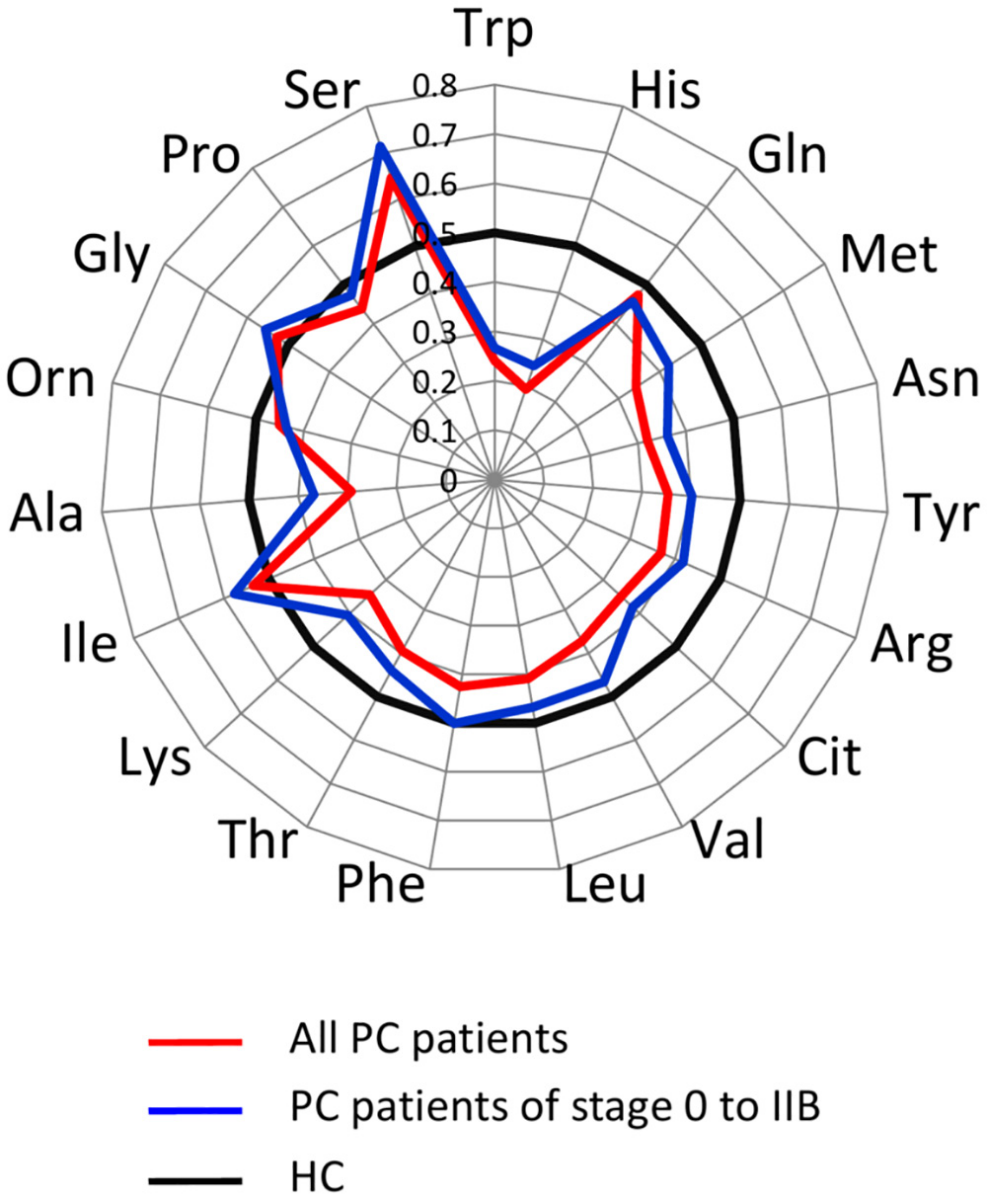
PFAA profiles of PC patients. The results of receiver–operator characteristic (ROC) curve analysis of PFAA profiles in the training set (120 PC and matching 600 HC). Axes show the AUC of ROC for each amino acid to discriminate patients from healthy controls. Black bold lines indicate the point where the AUC of ROC = 0.5.

We first measured the concentrations of 19 plasma amino acids in the training set by HPLC–ESI–MS and found significant increases in Ser concentrations and significant decreases in the concentrations of 14 amino acids (Thr, Asn, Pro, Ala, Cit, Val, Met, Leu, Tyr, Phe, His, Trp, Lys and Arg) in PC patients compared with HC subjects (*p* < 0.05) (Table 2, S1 Fig). The PFAA profiles were subjected to AUC of ROC analysis because the levels of significance are dependent on the sample size (Fig 2, S1 Table). Plasma Ser concentrations were especially higher, while Trp and His concentrations were particularly lower in PC patients compared with HC subjects. The PFAA profiles of PC patients with stage 0–IIB disease, as a resectable stage subgroup, were almost similar to those of all other PC patients.

**Table 2:** PFAA values (μmol/L) for patients with PC and healthy controls in the training set.

PFAA	HC (N = 600)	PC (N = 120)	
	Mean±SD (range)	Mean±SD (range)	p-value*
Thr	121.5 ± 24.3 (74.8–257.6)	112.8 ± 30.3 (54.2–215.5)	<0.001
Ser	109.1 ± 17.1 (47.9–177.6)	119.2 ± 22.5 (68.6–189.3)	<0.0001
Asn	45.4 ± 7.1 (28.6–94.6)	41.1 ± 7.4 (20.0–64.9)	<0.0001
Gln	575.2 ± 61.6 (327.6–787.1)	566.3 ± 87.0 (342.2–854.6)	≥0.1
Pro	141.4 ± 45.4 (57.1–360.8)	129.9 ± 36.1 (56.6–266.7)	<0.05
Gly	213.2 ± 53.1 (123.4–461.5)	219.1 ± 64.1 (102.5–515.3)	≥0.1
Ala	351.5 ± 72.2 (196.8–577.4)	296.5 ± 70.7 (157.0–517.7)	<0.0001
Cit	32.4 ± 6.9 (14.7–66.5)	28.5 ± 8.7 (9.8–51.5)	<0.0001
Val	220.9 ± 38.7 (126.6–358.3)	200.1 ± 42.9 (91.4–308.0)	<0.0001
Met	25.9 ± 4.7 (16.0–70.1)	23.4 ± 5.4 (13.0–42.5)	<0.0001
Ile	61.4 ± 13.8 (32.0–117.0)	63.2 ± 14.6 (30.1–109.3)	≥0.1
Leu	119.6 ± 22.4 (67.2–203.1)	111.6 ± 25.2 (47.1–188.9)	<0.01
Tyr	65.5 ± 12.1 (25.7–117.2)	58.9 ± 13.0 (33.3–103.3)	<0.0001
Phe	59.1 ± 8.0 (32.7–95.8)	57.2 ± 8.7 (34.8–84.2)	<0.01
His	80.3 ± 9.4 (51.6–120.7)	67.3 ± 11.9 (41.1–103.7)	<0.0001
Trp	56.7 ± 9.0 (10.6–88.9)	47.4 ± 10.1 (9.6–71.7)	<0.0001
Orn	53.6 ± 11.8 (25.1–136.1)	51.8 ± 15.6 (22.3–109.9)	≥0.1
Lys	192.3 ± 27.3 (118.9–276.1)	177.1 ± 39.7 (78.4–320.1)	<0.0001
Arg	94.2 ± 15.9 (50.3–149.0)	85.9 ± 22.3 (41.7–156.9)	<0.0001

*p values were calculated using the Mann–Whitney U-test.

### Multivariate PFAA index

For effective detection of PC patients, we calculated optimal PFAA indices by multiple logistic regression analysis. The AUC of ROC values obtained after LOOCV in the top 50 models were virtually the same (0.88–0.89). We evaluated a representative model composed of Ser, Asn, Ile, Ala, His, and Trp as the best model (Table 3). Then, additional logistic regression analyses adding BMI and/or smoking history into explanatory variables were performed to estimate the effects of potential confounding. No obvious elevation of significance was observed in each amino acid when those factors were added into the model, suggesting that the changes of the plasma level of those amino acids caused by PC were independent to BMI or smoking status of subjects (Table 3).

**Table 3:** Independence between PFAA used in the index and potential confounders such as BMI and smoking history.

Variable	P-value for a variable in logistic regression			
	Base (PFAA only)	+BMI	+Smoking	+BMI, Smoking
Ser	2.97x10^-9^	1.97x10^-8^	1.46x10^-9^	7.36x10^-9^
Asn	8.89x10^-2^	4.91x10^-2^	8.55x10^-2^	4.03x10^-2^
Ala	1.32x10^-3^	1.46x10^-2^	1.59x10^-3^	2.32x10^-2^
Ile	7.55x10^-9^	6.62x10^-10^	2.12x10^-8^	1.50x10^-9^
His	6.13x10^-11^	6.20x10^-11^	1.16x10^-10^	1.54x10^-10^
Trp	2.66x10^-4^	7.42x10^-4^	4.55x10^-4^	1.42x10^-3^
BMI		5.05x10^-4^		1.26x10^-4^
Smoking			9.19x10^-1^	9.77x10^-1^

**Fig 3:**
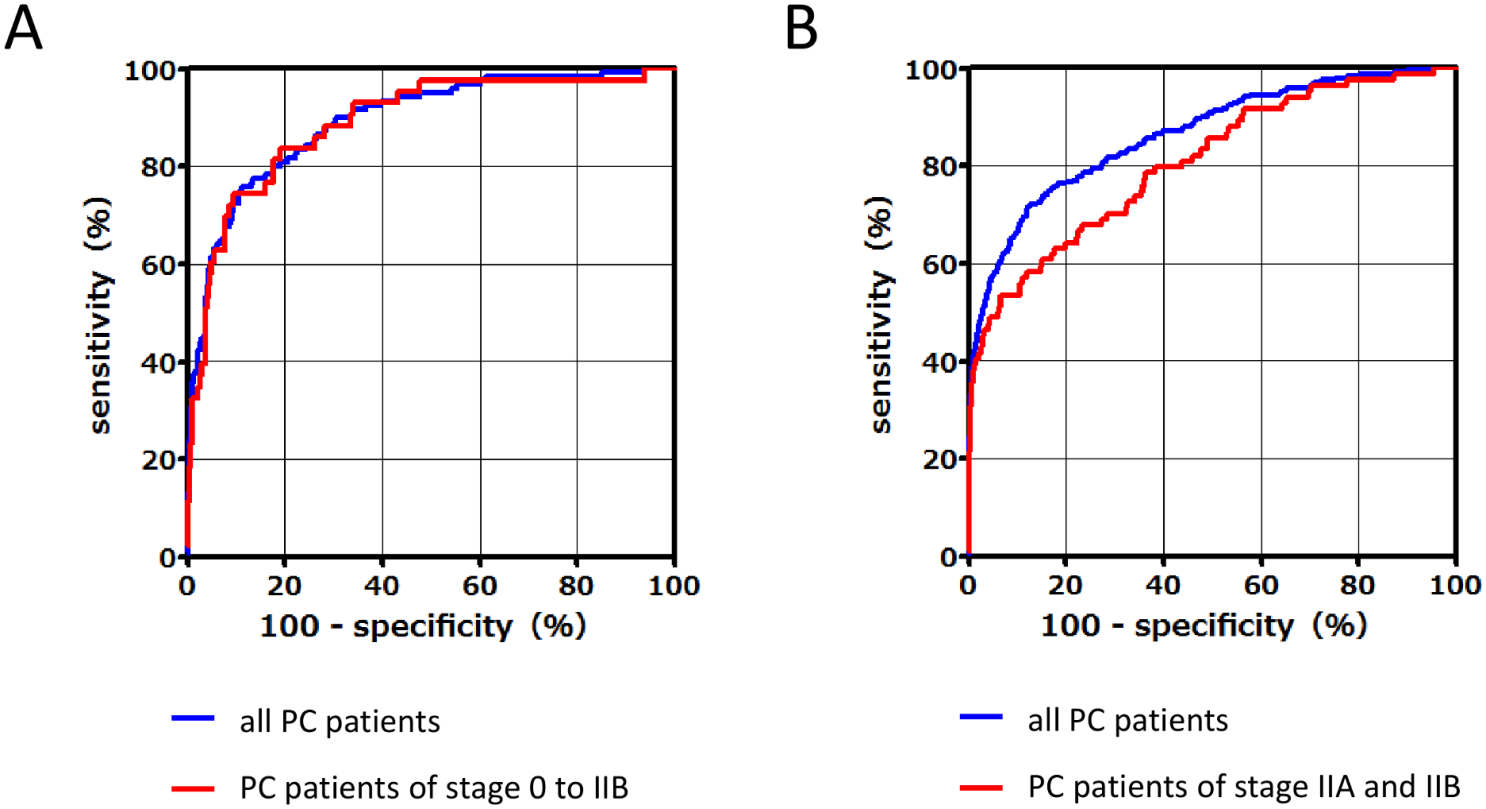
ROC curves of the PFAA index of PC patients compared with those of healthy controls in the training set (120 PC and matching 600 HC) (A) and the validation set (240 PC and 7772 HC) (B).

In terms of discriminating PC patients from control subjects, ROC curves for the PC vs. HC or CP subgroups between the training set and validation set were calculated (Fig 3A and 3B, respectively). In the training set, the AUCs of the PFAA indices for detection of PC patients vs. HC subjects was 0.89 (95% CI, 0.86–0.93) among all PC patients and 0.89 (95% CI, 0.83–0.95) among PC patients with stage 0–IIB disease. The sensitivities of the PFAA indices at 95% and 80% specificity were 60.0% and 82.5%, respectively, for all PC patients, and 53.5% and 83.7% for PC patients with stage 0–IIB disease, respectively (Table 4). In the validation set, the AUC of the PFAA index was 0.86 (95% CI, 0.84–0.89) for all PC patients and 0.81 (95% CI, 0.75–0.86) for PC patients with stages IIA and IIB disease (Fig 3B). The sensitivities of the PFAA indices at a specificity of 95% and 80% were 57.5% and 76.7% for all PC patients, and 48.8% and 64.3% for PC patients with stage IIA and IIB disease (Table 4). The AUC of the PFAA index for detection of PC vs. CP was 0.87 (95% CI, 0.80–0.93) for all PC patients and the false-positive rates at 95% and 80% specificity were 7.1% and 25.0%, respectively (Table 4). S2 Fig shows box plots of PFAA index in HC, PC, and CP in the validation set.

**Table 4:** Discrimination performance of PFAA index.

		PFAA index^a^	
	Specificity (%)	95	80
Training set:	AUC	0.89	
	(95% confidence interval)	(0.86–0.93)	
	Sensitivity (%)	60.0	82.5
	Sensitivity for stage 0 to IIB PC (%)	53.5	83.7
Validation set:	AUC	0.86	
	(95% confidence interval)	(0.84–0.89)	
	Sensitivity (%)	57.5	76.7
	Sensitivity for stage IIA and IIB PC (%)	48.8	64.3
	False-positive rate in CP (%)	7.1	25.0

^**a**^: Following PFAAs was used as variables: Ser, Asn, Ile, Ala, His, and Trp.

We confirmed that the variance inflating factor (VIF), the maximum of the diagonal element of the inverse matrix of correlation coefficient matrix, of all the top 50 models not to choose the inappropriate models showing multicolinearity. All the models passed the test, that is, VIFs were less than 10. Most of all, VIF of the representative model was 1.70, suggesting that no multicolinearity occured.

**Fig 4:**
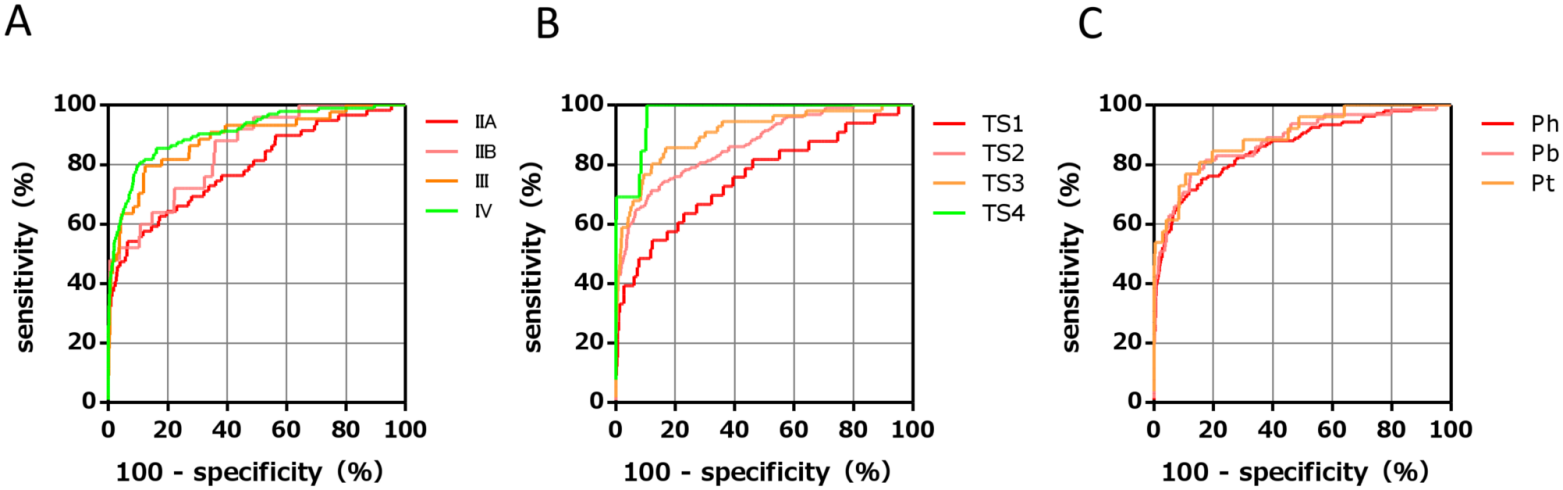
ROC curves of the PFAA index with different tumor stages, sizes, and locations. (A) ROC curves of the PFAA index in stage IIA (red), stage IIB (pink), stage III (orange), and stage IV (yellow–green), respectively. (B) ROC curves in TS1 (red), TS2 (pink), TS3 (orange), and TS4 (yellow–green), respectively. TS1 ≤ 2.0 cm, 2.0 cm < TS2 ≤ 4.0 cm, 4.0 cm < TS3 ≤ 6.0 cm, and TS4 > 6.0 cm. (C) ROC curves in the pancreatic head (red), body (pink), and tail (orange), respectively.

**Fig 5:**
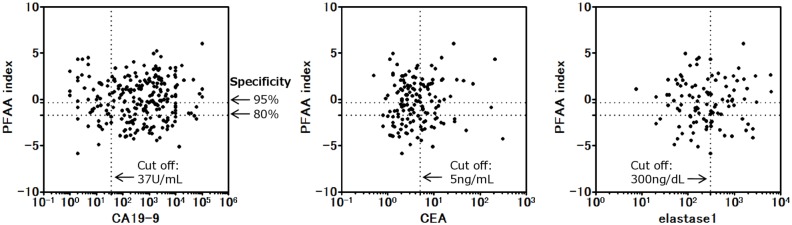
Correlation of PFAA index and other biomarkers (CA19-9, CEA, and elastase 1). The dotted line shows the cut-off of each biomarker or PFAA index. For data analysis, the upper normal limits of CA19-9, CEA, and elastase-1 were defined as 37 U/mL, 5 ng/dL, and 300 ng/dL, respectively. There were no significant correlations between each biomarker and the PFAA index.

Furthermore, subgroup analysis was performed for tumor stage, size, and location in the pancreas. The AUC of the PFAA index according to tumor stage was as follows: 0.79 (95% CI, 0.72–0.86) for stage IIA, 0.85 (95% CI, 0.77–0.92) for stage IIB, 0.88 (95% CI, 0.83–0.94) for stage III, and 0.91 (95% CI, 0.88–0.94) for stage IV (Fig 4A). The AUC of the PFAA index according to tumor size was as follows: 0.76 (95% CI, 0.66–0.86) for TS1, 0.87 (95% CI, 0.83–0.90) for TS2, 0.91 (95% CI, 0.86–0.95) for TS3, and 0.97 (95% CI, 0.95–1.00) for TS4 (Fig 4B). The AUC of the PFAA index according to tumor location was as follows: 0.86 (95% CI, 0.82–0.90) for the pancreatic head, 0.88 (95% CI, 0.83–0.93) for the pancreatic body, and 0.90 (95% CI, 0.83–0.96) for the pancreatic tail (Fig 4C). In addition, we evaluated the correlations between PFAA index values and other biomarkers (i.e., CA19-9, CEA, and elastase-1) because the combinatorial use of multiple independent tumor markers is effective to detect PC. There were no significant correlations between the PFAA indices and levels of CA19-9 (r = 0.075, *p* = 0.247), CEA (r = −0.005, *p* = 0.957), or elastase-1 (r = 0.009, *p* = 0.351) in PC (Fig 5, S2 Table).

## Discussion

Dysregulation of PFAA content in PC has been investigated in several recent studies using metabolomics or amino acid analysis [21][22][23]. However, specific PFAA profiles in PC, particularly at resectable stages, remain unconfirmed because of the relatively small number of PC patients and control subjects used in these studies. Therefore, we measured fasting PFAA concentrations in a large-scale study of 360 PC patients and 8372 control subjects to identify specific PFAA profiles in PC patients as compared with a gender- and age-matched training set (PC120, HC600) (Fig 2). In addition, a similar PFAA profile was observed in patients with stage 0–IIB disease, which accounted for 35.8% of the PC patients included in this study. (Fig 2). As shown in Table 2, the plasma concentrations of several amino acids were significantly altered in PC patients, which were in accordance with the PFAA profiles of five types of cancer reported by Miyagi et al. [14], although plasma His and Trp concentrations were particularly decreased, while Ser concentrations were notably increased (Fig 2). Furthermore, we developed a PFAA index using a training set composed of six amino acids that were clearly characteristic of the amino acid profile in PC. We demonstrated that this index can be used to efficiently differentiate not only progressive PC but also operable PC, from stage IIA and IIB disease in an independent validation set (Table 4). Moreover, we also showed that the PFAA index hardly detects chronic pancreatitis (Table 4).

PFAA profiles of PC patients have been reported in several previous studies, among which, several amino acid profiles were similar, although there were some obvious discrepancies [21][22][23] For example, we found a significant increase in plasma Ser concentrations in PC, while this trend was not observed in other studies. In addition, there were discrepancies in Asn, Gln, Met, Ile, Phe, Leu, and Pro levels. In contrast, these previous studies commonly reported a significant decrease in Thr concentrations, while changes in Arg, Cit, and Trp concentrations were not determined in one study, and significant decreases in these amino acids in PC were observed in our study as well as two others. We considered several reasons for these discrepancies. First, these previous studies included relatively small numbers of subjects compared with the present study, which included the largest number of subjects to date. In this study, the PFAA index was robust and the AUC barely decreased even with the validation set because it was developed based on a training set with an adequate sample size. Second, differences between our results and those of other studies may have occurred because of variations in sample preparation conditions and analytical methods. Third, PFAA profiles exhibit diurnal fluctuations [24][25][26] and change according to circadian rhythms [27][28] because they are largely dependent on recent meals, even among healthy subjects [24][25][26][29]. Furthermore, leaving collected blood samples at room temperature is known to alter plasma amino acid concentrations [30]. To overcome this confounding factor, all participating facilities in this study used the same protocol, in which blood was drawn in the morning before breakfast after overnight fasting and the collected samples were quickly cooled to prevent alterations in amino acid concentrations because of enzymatic reactions. Therefore, the acquired samples were of high quality and the extracted data accurately reflects in vivo amino acid profiles during fasting. Furthermore, determination of amino acid concentrations using HPLC–ESI–MS in this study was calculated not as a semiquantified value using metabolomics as in previous studies but rather as directly quantified absolute concentrations by creating a calibration curve from the peak area of standard references of each amino acid [19]. These measurements were highly accurate and precise to guarantee validation, reproducibility, and limited daily error [19]. Thus, the findings of this study may more clearly demonstrate profile characteristics in comparison with those demonstrated by previous studies. The use of multivariate analysis of markers for PC has also been reported [23, 31]. For example, Kobayashi et al. [23] constructed a multiple logistic regression model using a 43-case training set with the concentrations of four metabolites selected as variables from data comprehensively semiquantified from metabolite concentrations by GC–MS. Meanwhile, of the four selected metabolites, xylitol is a food-derived substance that is present at very low concentrations in healthy individuals [32][33]. However, it was unclear whether these concentrations are physiologically maintained at certain levels in vivo. Furthermore, Leichtle et al. [31] constructed a combined metabolite panel to discriminate PC from CP and HC using aspartic acid (Asp) and CA19-9 as variables. However, the plasma Asp concentration tends to be comparatively low and an analytical variability of >25%, as reported elsewhere [34]. In the present study, the PFAA index was constructed with only amino acids with moderate to high plasma concentrations to secure measurement precision. Therefore, we believe that the PFAA index offers a high discriminatory ability without being influenced by measurement errors. Because amino acid analysis is widely used clinically, the PFAA index is likely to be quickly verified and we suspect its use will be widespread in the near future. Meanwhile, genetic, racial, and geographical elements may also be factors impacting these differences, which should be clarified in future research.

There are several possible mechanisms that may influence PFAA profiles in cancer patients. First, previous studies have demonstrated marked metabolic changes in local cancer, including varied amino acid profiles and different expression of amino acid transporter in cancer cells compared with healthy cells [10][35]. For example, L-neutral amino acid transporter 1 (LAT1) is strongly expressed in PC cells [36]. With respect to Ser, de novo Ser biosynthesis is upregulated in cancer cells and Ser acts as an allosteric activator of pyruvate kinase isozyme M2 [37]. This characteristic may be related to factors that also increase plasma Ser concentrations. A second possible mechanism is the induction of remote organ metabolic changes caused by factors emitted from cancer cells. For example, Luo et al. [38] reported that HMGB-1 secreted by cancer cells caused the breakdown of remote muscle tissue proteins into amino acids, some of which leak out into the blood, thereby altering the PFAA profile. A third possible mechanism is involvement of the immune system. For example, plasma concentrations of Trp have been correlated with common metabolic changes, both in our study and a previous study that investigated Trp levels in five different types of cancer [14]. Expression of indoleamine 2,3-dioxygenase (IDO), which is involved in the kynurenine metabolic pathway, is induced in various types of cancer (cancer cells or immune cells) and known to play an important role in immunosuppression [39]. IDO is also known to be overexpressed in PC cells [40]. Thus, several points regarding the mechanisms behind changes in PFAA profiles in PC remain unclear; thus, further research is needed to clarify these issues.

Recently, Mayers et al. [41] reported that branched-chain amino acid (BCAA) serum levels are elevated 2–5 years before the onset of carcinogenesis in PC, suggesting that BCAA elevation is an independent risk factor for PC. However, BCAA levels return to normal levels within the 2 years before confirmation of cancer. In addition, the results of a mouse study indicated that the period of BCAA elevation was bell-shaped and only temporary. In our study, we presented the PFAA profiles of definitively diagnosed patients after cancer detection via diagnostic imaging. Among cases with resectable stage disease (up to stage IIB), there were no significant changes in BCAA concentrations compared with control subjects. In cases of advanced cancer, Leu and Val concentrations were decreased. Our study data identified characteristics of PC phases that are readily confirmed by currently available imaging modalities; thus, it is possible that these characteristics differed among the main stages of microcarcinoma or before the onset of carcinogenesis. Because we did not examine PFAA concentrations before carcinogenesis in this study, future studies are needed to accurately identify BCAA dynamics from before PC onset to carcinogenesis. However, the abovementioned studies found that metabolic changes alter systemic amino acid profiles together with changes in plasma BCAA concentrations in the precancerous phase or extremely early stages of PC [41]. Therefore, the observed changes in PFAA profiles of the patients with PC lesions, which could be diagnosed via imaging and considered for resection in our study, may also have been caused by systemic metabolic changes.

Currently, CA19-9 is the most widely used marker to predict PC treatment outcome and post-treatment prognosis [42]. However, CA19-9 is not synthesized by patients classified as Lewis blood group Le^a-b-^, which accounts for 10% of cases; therefore, this marker may not be elevated in some patients, even those with advanced stage PC [43]. CEA is widely used as a prognostic marker in gastrointestinal cancers; however, its sensitivity and specificity for PC are poor [44]. The pancreatic enzyme elastase-1, which is thought to increase with pancreatitis caused by pancreatic duct stenosis, has been demonstrated as an effective early diagnostic marker [45]. In the present study, we found no correlations between the PFAA index and CA19-9, CEA, or elastase-1 levels (Fig 5). Thus, when used concurrently, the PFAA index with CA19-9, CEA, or elastase-1 may complement each other in order to more accurately detect PC. However, CA19-9 and elastase-1 were not measured in the HC subjects in this study. Thus, to confirm the comparison of accurate discriminatory ability and synergetic effect with these markers, further studies are needed. The discriminatory ability of the PFAA index was shown to be high even for small pancreatic tumors of TS-1 according to subgroup analysis (ROC_AUC = 0.76) (Fig 4B). We also found that the PFAA index was not dependent on the location of the pancreatic tumor (Fig 4C). Although general abdominal ultrasonography is used to diagnose PC in the initial phase, it is difficult to image small tumors or lesions in the pancreatic tail or uncinate process using this modality. Our results suggested that the proposed PFAA index developed in this study offers the same sensitivity without depending on tumor location. Therefore, combinatorial use of abdominal ultrasonography and the PFAA index may be a good marker to increase the detection rate of lesions of the pancreatic tail and uncinate process. In this study, the training set and validation set were divided chronologically. As a result, no early cases of stage I or less were included in the validation set that occurred chronologically later in time. The fact that the discriminatory ability of the PFAA index for early stage cases of stage I or less remains unknown is a limitation of this study. However, this study was cross-sectional; therefore, we cannot exclude the possibility of reverse causation or residual confounding from complications such as diabetes or indigestion. In our future work, we plan to demonstrate the clinical significance of our proposed PFAA index and confirm its ability to discriminate the early stages of PC and the association of the PFAA index with the complications of PC.

## Conclusions

In this study, we successfully developed a novel PFAA index using fasting PFAA profiles to discriminate PC patients from control subjects, and validated the index in an independent large validation set, although the study was cross-sectional and the reversal causality, including symptoms associated with PC or complications, cannot be ruled out. Additional studies with larger patient cohort that include patients with early stage PC are also required. However, we believe the PFAA index will help to improve the early detection of PC in patients with asymptomatic and resectable stage disease.

## Supporting Information

S1 Fig Box plot of amino acid values (μmol/L) for patients with pancreatic cancer (n = 120) and healthy controls (n = 600). Box plots display the 10th, 25th, 50th (median), 75th, and 90th percentiles. P values were calculated by the Mann-Whitney test.
(DOCX)

S2 Fig Box plots of PFAA index in patients with pancreatic cancer (n = 240), pancreatitis (n = 28), and healthy controls (n = 7772). Box plots display the 10th, 25th, 50th (median), 75th, and 90th percentiles. Kruskal-Wallis test with Dunn’s post-test, PC versus HC, CP, ***p<0.001.
(DOCX)

S1 Table AUCs of ROC of each amino acid concentration for discrimination of cancer patients from controls. (DOC)

S2 Table Pearson's coefficient of correlations (r-values) for relationship between PFAA index and other biomarkers with different stages. (DOC)
