# Supplementary material for: A Novel Multivariate Index for Pancreatic Cancer Detection Based On the Plasma Free Amino Acid Profile
Source: PLoS One. 2015 Jul 2;10(7):e0132223. doi: 10.1371/journal.pone.0132223 (PMC4489861; doi:10.1371/journal.pone.0132223)

**S1 Fig. Box plot of amino acid values (μmol/L) for patients with pancreatic cancer (n=120) and healthy controls (n=600).** Box plots display the 10th, 25th, 50th (median), 75th, and 90th percentiles. P values were calculated by the Mann-Whitney test.


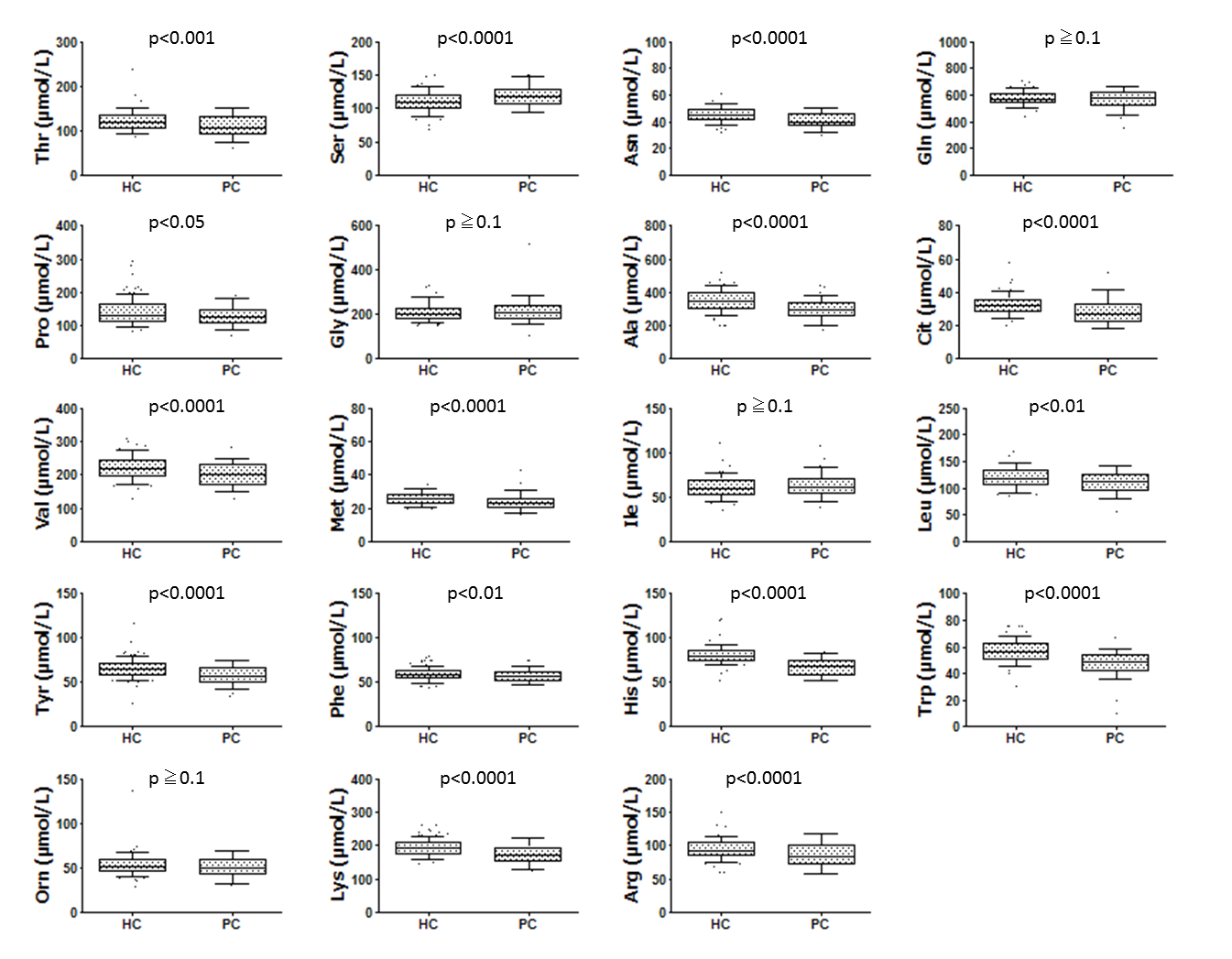

Supplement: S1 Fig — Box plots display the 10th, 25th, 50th (median), 75th, and 90th percentiles. P values were calculated by the Mann-Whitney test. (DOCX) [file pone.0132223.s001.docx]
