# Supplementary material for: A Novel Multivariate Index for Pancreatic Cancer Detection Based On the Plasma Free Amino Acid Profile
Source: PLoS One. 2015 Jul 2;10(7):e0132223. doi: 10.1371/journal.pone.0132223 (PMC4489861; doi:10.1371/journal.pone.0132223)

**S2 Fig. Box plots of PFAA index in patients with pancreatic cancer (n=240), pancreatitis (n=28), and healthy controls (n=7772).** Box plots display the 10th, 25th, 50th (median), 75th, and 90th percentiles. Kruskal-Wallis test with Dunn’s post-test, PC versus HC, CP, ***p<0.001.


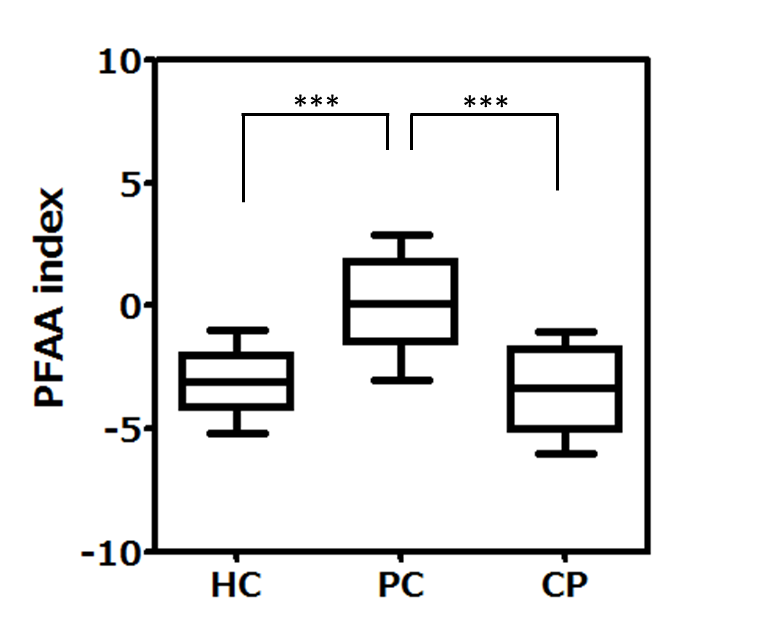

Supplement: S2 Fig — Box plots display the 10th, 25th, 50th (median), 75th, and 90th percentiles. Kruskal-Wallis test with Dunn’s post-test, PC versus HC, CP, ***p<0.001. (DOCX) [file pone.0132223.s002.docx]
