# Supplementary material for: A Novel Multivariate Index for Pancreatic Cancer Detection Based On the Plasma Free Amino Acid Profile
Source: PLoS One. 2015 Jul 2;10(7):e0132223. doi: 10.1371/journal.pone.0132223 (PMC4489861; doi:10.1371/journal.pone.0132223)
Supplement: S1 Table — (DOC) [file pone.0132223.s003.doc]

**S1 Table. AUCs of ROC of each amino acid concentration for discrimination of cancer patients from controls**

|  | **all PC** | **PC at stage 0 to IIB** |
| --- | --- | --- |
|  | **AUC (95%CI)** | **AUC (95%CI)** |
| Thr | 0.39 (0.33–0.46) | 0.44 (0.33-0.55) |
| Ser | 0.65 (0.59–0.70) | 0.71 (0.62-0.80) |
| Asn | 0.32 (0.26–0.38) | 0.36 (0.26-0.46) |
| Gln | 0.47 (0.41–0.54) | 0.46 (0.35-0.56) |
| Pro | 0.44 (0.38–0.49) | 0.47 (0.38-0.56) |
| Gly | 0.53 (0.47–0.59) | 0.56 (0.45-0.66) |
| Ala | 0.29 (0.24–0.34) | 0.37 (0.28-0.46) |
| Cit | 0.35 (0.29–0.41) | 0.38 (0.28-0.48) |
| Val | 0.37 (0.32–0.43) | 0.47 (0.37-0.56) |
| Met | 0.34 (0.28–0.40) | 0.42 (0.31-0.53) |
| Ile | 0.54 (0.48–0.59) | 0.58 (0.49-0.66) |
| Leu | 0.41 (0.35–0.47) | 0.47 (0.37-0.57) |
| Tyr | 0.35 (0.30–0.41) | 0.40 (0.31-0.49) |
| Phe | 0.43 (0.37–0.48) | 0.50 (0.41-0.59) |
| His | 0.19 (0.15–0.24) | 0.24 (0.16-0.33) |
| Trp | 0.24 (0.19–0.29) | 0.27 (0.18-0.35) |
| Orn | 0.45 (0.39–0.51) | 0.44 (0.34-0.54) |
| Lys | 0.34 (0.29–0.40) | 0.41 (0.30-0.51) |
| Arg | 0.37 (0.31–0.43) | 0.42 (0.31-0.53) |
