# Supplementary material for: A Novel Multivariate Index for Pancreatic Cancer Detection Based On the Plasma Free Amino Acid Profile
Source: PLoS One. 2015 Jul 2;10(7):e0132223. doi: 10.1371/journal.pone.0132223 (PMC4489861; doi:10.1371/journal.pone.0132223)
Supplement: S2 Table — (DOC) [file pone.0132223.s004.doc]

**S2 Table. Pearson's coefficient of correlations (r-values) for relationship between PFAA index and other biomarkers with different stages.**

| **parameter** | **stage** | **PFAA index** | |
| --- | --- | --- | --- |
|  |  | **r-value** | **p-value** |
| CA19-9  (U/mL) | ALL | 0.075 | 0.247 |
|  | IIA | 0.141 | 0.288 |
|  | IIB | 0.259 | 0.212 |
|  | III | -0.013 | 0.934 |
|  | IV | 0.021 | 0.834 |
| CEA  (ng/mL) | ALL | -0.005 | 0.957 |
|  | IIA | -0.207 | 0.138 |
|  | IIB | -0.154 | 0.493 |
|  | III | 0.285 | 0.199 |
|  | IV | 0.182 | 0.275 |
| elastase-1  (ng/dL) | ALL | 0.009 | 0.351 |
|  | IIA | -0.091 | 0.570 |
|  | III | 0.050 | 0.826 |
|  | IV | 0.191 | 0.257 |
